# Relationship between Non-Alcoholic Fatty Liver Disease and Psoriasis: A Novel Hepato-Dermal Axis?

**DOI:** 10.3390/ijms17020217

**Published:** 2016-02-05

**Authors:** Alessandro Mantovani, Paolo Gisondi, Amedeo Lonardo, Giovanni Targher

**Affiliations:** 1 Section of Endocrinology, Diabetes and Metabolism, Department of Medicine, University and Azienda Ospedaliera Universitaria Integrata of Verona, Piazzale Stefani, 1, Verona 37126, Italy; alessandro.mantovani24@gmail.com; 2 Section of Dermatology, Department of Medicine, University and Azienda Ospedaliera Universitaria Integrata of Verona, Piazzale Stefani, 1, Verona 37126, Italy; paolo.gisondi@univr.it; 3 Outpatient Liver Clinic and Division of Internal Medicine—Department of Biomedical, Metabolic and Neural Sciences, NOCSAE, University of Modena and Reggio Emilia and Azienda USL Modena, Baggiovara, Modena 41126, Italy; a.lonardo@libero.it

**Keywords:** nonalcoholic fatty liver disease, NAFLD, nonalcoholic steatohepatitis, management, psoriasis

## Abstract

Over the past 10 years, it has become increasingly evident that nonalcoholic fatty liver disease (NAFLD) is a multisystem disease that affects multiple extra-hepatic organ systems and interacts with the regulation of several metabolic and immunological pathways. In this review we discuss the rapidly expanding body of clinical and epidemiological evidence supporting a strong association between NAFLD and chronic plaque psoriasis. We also briefly discuss the possible biological mechanisms underlying this association, and discuss treatment options for psoriasis that may influence NAFLD development and progression. Recent observational studies have shown that the prevalence of NAFLD (as diagnosed either by imaging or by histology) is remarkably higher in psoriatic patients (occurring in up to 50% of these patients) than in matched control subjects. Notably, psoriasis is associated with NAFLD even after adjusting for metabolic syndrome traits and other potential confounding factors. Some studies have also suggested that psoriatic patients are more likely to have the more advanced forms of NAFLD than non-psoriatic controls, and that psoriatic patients with NAFLD have more severe psoriasis than those without NAFLD. In conclusion, the published evidence argues for more careful evaluation and surveillance of NAFLD among patients with psoriasis.

## 1. Introduction

Psoriasis is a chronic, immune-mediated, inflammatory skin disease that affects approximately 2%–3% of the adults in the general population of Western countries [1,2]. This disease is known for its typical cutaneous manifestations; described as well-demarcated, erythematous oval plaques with adherent silvery scales. However, recent studies have also linked psoriasis with multiple comorbid conditions, including arthritis, uveitis, inflammatory bowel diseases, depression, osteoporosis, cardiovascular disease and metabolic syndrome [3].

In parallel, nonalcoholic fatty liver disease (NAFLD) is the most frequent liver disease worldwide, affecting an estimated 30% of the adult population in developed countries [4,5]. NAFLD and the metabolic syndrome are mutually and bi-directionally associated, as these two pathologic conditions share insulin resistance as a common pathophysiological mechanism [6,7,8]. NAFLD encompasses a spectrum of pathologic conditions ranging from simple steatosis to nonalcoholic steatohepatitis ((NASH) featuring steatosis associated with inflammatory changes, hepatocellular ballooning and pericellular fibrosis), to advanced fibrosis and cirrhosis. NAFLD is projected to become the most common indication for liver transplantation in the United States by 2030 [5,9]. However, over the past 10 years, it has become increasingly clear that NAFLD is not only associated with increased liver-related mortality or morbidity, but also is a multisystem disease affecting a variety of extra-hepatic organ systems, including the heart and the vascular system [9,10]. Cardiovascular disease represents the primary cause of mortality in NAFLD patients [9,10].

In this updated review we will discuss the clinical evidence supporting a link between NAFLD and chronic plaque psoriasis, and the putative mechanisms underlying this association. We will also briefly discuss some of the therapeutic options for psoriasis that may influence NAFLD development and progression. We extensively searched PubMed database to identify original articles published through December 31st 2015, using the following key-words “nonalcoholic fatty liver disease” or “NAFLD” combined with “chronic plaque psoriasis”, “psoriasis” or “psoriatic treatment”.

## 2. Epidemiology, Clinical Manifestations and Pathogenesis of Psoriasis

Psoriasis is a chronic, recurrent, immune-mediated inflammatory disease of the skin, affecting approximately 2%–3% of the general adult population in many parts of the world [1]. The prevalence of this disease in adults ranges from approximately 1% (United States) to 8.5% (Norway). The incidence estimate varies from approximately 80/100,000 person-years (United States) to 230/100,000 person-years (Italy) [1]. Epidemiological studies suggest that the prevalence of psoriasis varies according to increasing age and is more common in countries more distant from the equator [1]. However, additional studies are needed to better understand the epidemiology of psoriasis and trends in incidence over time.

**Figure 1:**
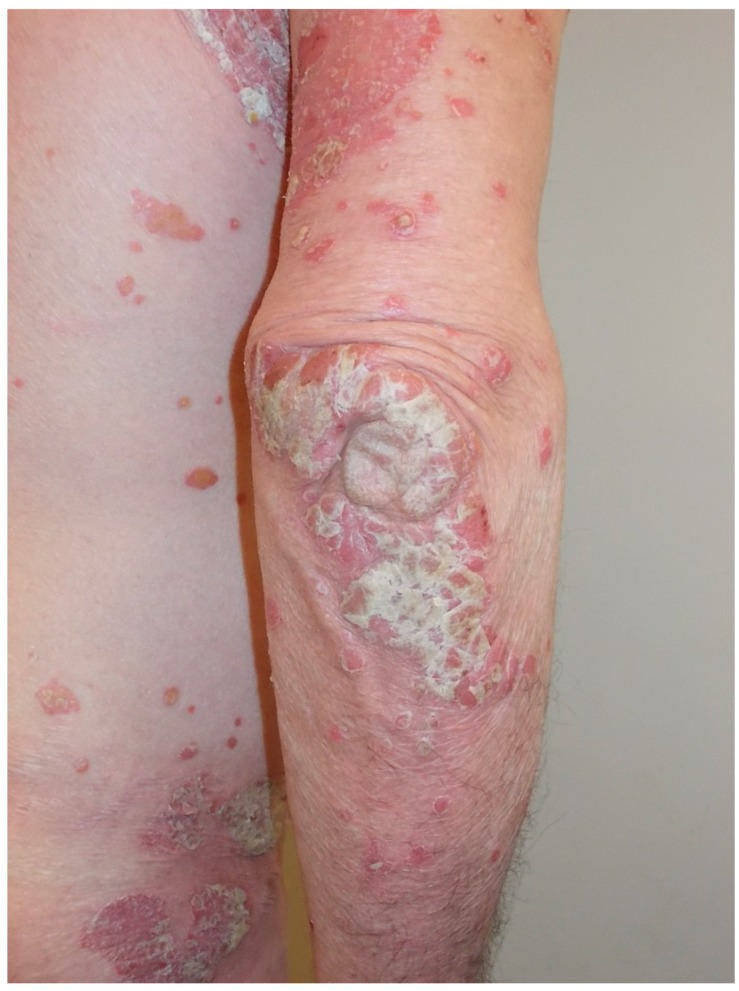
Psoriatic lesions on the elbows.

Psoriasis manifests as raised, irregularly round and well-demarcated erythematous lesions that are usually covered by silver scales (Figure 1).

Psoriatic lesions are distributed symmetrically on the scalp, elbows, knees, lumbo-sacral area and in the body folds. Psoriatic lesions are frequently symptomatic with pruritus by far the most bothersome skin symptom reported by the patients, even for those with limited disease, followed by scaling and flaking. Psoriasis may have a negative impact on the physical, emotional and psychosocial wellbeing of affected patients. About one third of patients have symptoms of arthritis, which might be very disabling in the more severe cases [11]. Psoriasis is also frequently associated with multiple metabolic co-morbidities, including abdominal overweight or obesity, type 2 diabetes, metabolic syndrome and NAFLD [3,12,13].

The exact aetiology of psoriasis is largely unknown. However, strong evidence indicates that psoriasis is a chronic inflammatory skin disease, occurring against a predisposing genetic background. The pathogenesis of psoriasis is complex, with a combination of genetic and environmental factors playing an integrated role [2]. The contribution of genetic factors to the pathogenesis of psoriasis is extensive, with the human leukocyte antigen (HLA)-C*06 showing the most significant association, although genome-wide association studies have identified more than 35 psoriasis risk gene regions primarily involved in innate and adaptive immunity [14]. A deregulated cytokine network occurs in psoriasis, leading to the release of multiple pro-inflammatory mediators from immune cells, which in turn induce increased keratinocyte proliferation [15]. Psoriasis is thought to be a T cell-driven disease, with the Th1 and Th17 cell populations playing a major role. These immune cells produce a variety of pro-inflammatory cytokines, including tumour necrosis factor (TNF)-α, interleukin (IL)-6, IL-17, IL-22 and interferon-gamma, resulting in abnormal differentiation and proliferation of keratinocytes, blood vessels dilatation and inflammatory infiltration of leukocytes into the dermis and epidermis [15,16]. A number of environmental factors have been also identified as possible triggers of psoriasis, including physical traumas (known as Koebner’s phenomenon), bacterial infections, stressful life events or use of some drugs, such as interferon α and lithium salts [2,15]. However, more precise identification of genetic and environmental factors that are potentially involved in the development of psoriasis will help to better elucidate the pathogenesis of this disease and identify new targets for a more specific and effective treatment.

## 3. Epidemiological Evidence Linking Nonalcoholic Fatty Liver Disease (NAFLD) to Psoriasis

Given the strong relationship of the metabolic syndrome with both psoriasis [3,12,13] and NAFLD [4,5,6], it is perhaps not surprising that these two latter diseases may coexist within the same individual.

In a case report published in 2001 Lonardo *et al*. [17] were the first to describe three cases of concurrent psoriasis vulgaris and NASH, diagnosed on biopsy. All patients were obese and had other features of the metabolic syndrome. Similarly, Matsumoto *et al*. [18] described a case of a young obese psoriatic man with NASH that improved after hypocaloric diet.

As detailed in Table 1, after these pioneering case reports, multiple observational (cross-sectional and case-control) studies have recently assessed whether NAFLD (as diagnosed either by ultrasonography or by histology) is associated with psoriasis [19,20,21,22,23,24,25,26,27].

**Table 1:** Principal studies examining the relationship between NAFLD and psoriasis (ordered by publication year).

Authors, Year (Reference)	Study Characteristics	NAFLD Diagnosis	Main Findings
Gisondi *et al.* 2009 [19]	Cross-sectional: 130 consecutive Italian patients with chronic plaque psoriasis and 260 healthy controls matched for age, sex and BMI	Ultrasonography	Prevalence of NAFLD was remarkably higher in psoriatic patients than in matched controls (47% *vs.* 28%; *p* < 0.001). Patients with psoriasis and NAFLD were more likely to have metabolic syndrome and had higher serum C-reactive protein concentrations and greater severity of psoriasis according to PASI score than those with psoriasis alone. At multivariate linear regression analysis, NAFLD was associated with higher PASI score (standardized β coefficient 0.19, *p* = 0.03), independent of age, sex, BMI, psoriasis duration and alcohol consumption
Miele *et al.* 2009 [20]	Retrospective, case-control: 142 Italian patients with psoriasis and 125 non-psoriatic patients with biopsy-proven NAFLD comparable for age and BMI	Ultrasonography and biopsy	Prevalence of NAFLD was 59.2% in the cohort of psoriatic patients. In these patients NAFLD was significantly associated with metabolic syndrome and psoriatic arthritis. Compared with the non-psoriatic NAFLD cohort, psoriatic patients with NAFLD were likely to have more severe NAFLD reflected by either non-invasive NAFLD Fibrosis score or AST/ALT ratio >1
Madanagobalane *et al.* 2012 [21]	Cross-sectional: 333 Indian psoriatic patients and 330 controls matched for age, sex and BMI	Ultrasonography and liver enzymes	Prevalence of NAFLD was higher in psoriatic patients than in matched controls (17.4% *vs.* 7.9%; *p* < 0.005). Psoriatic patients with NAFLD had more severe psoriasis than those without NAFLD. In a subset of participants, psoriatic patients had more severe forms of NAFLD than non-psoriatic patients with NAFLD (as estimated by non-invasive fibrosis markers)
van der Voort *et al.* 2014 [22]	Cross-sectional: population-based cohort of 2292 Dutch elderly participants (the Rotterdam Study)	Ultrasonography	Prevalence of psoriasis was 5.1% (by a validated algorithm). Prevalence of NAFLD was higher in psoriatic patients than in participants without psoriasis (46.2% *vs.* 33.3%, *p* = 0.005). Psoriasis was associated with NAFLD (OR 1.70, 95% CI 1.1–2.6, *p* = 0.01), independent of age, sex, alcohol consumption, pack-years and smoking status, metabolic syndrome, and serum ALT levels
van der Voort *et al.* 2015 [23]	Cross-sectional: population-based cohort of 1535 elderly participants (the Rotterdam Study) of whom 74 (4.7%) had psoriasis	Ultrasonography and transient elastography (Fibroscan)	Prevalence of NAFLD was higher in subjects with psoriasis than in those without psoriasis (44.3% *vs.* 34%, *p* < 0.05). Moreover, prevalence of advanced liver fibrosis was 8.1% in psoriatic patients compared with 3.6% in the control group (*p* < 0.05). Multivariate logistic regression analysis revealed that the risk of advanced liver fibrosis remained higher in psoriatic patients after adjustment for age, sex, alcohol consumption, serum ALT levels, presence of metabolic syndrome and hepatic steatosis (OR 2.57, 95% CI 1.0–6.6)
Gisondi *et al.* 2015 [24]	Cross-sectional: 124 Italian patients with psoriasis and 79 healthy controls	Ultrasonography	Prevalence of NAFLD was higher in psoriatic patients than in controls (44% *vs.* 26%, *p* < 0.001). NAFLD fibrosis score was also higher in psoriatic patients (*p* < 0.001). Multivariate regression analysis revealed that psoriasis was associated with higher NAFLD fibrosis score, independent of age, sex, BMI, hypertension and pre-existing diabetes
Abedini *et al.* 2015 [25]	Cross-sectional: 123 Iranian patients with psoriasis and 123 healthy controls matched by age, sex and BMI	Ultrasonography	Prevalence of NAFLD was higher in psoriatic patients than in matched controls (65.6% *vs.* 35%, *p* < 0.01). Multivariate logistic regression analysis revealed that PASI score, waist circumference, hypertension and serum aminotransferase levels independently predicted the ultrasonographic severity of NAFLD
Roberts *et al.* 2015 [26]	Cross-sectional: 103 United States adult patients with a diagnosis of psoriasis or psoriatic arthritis	Ultrasonography and biopsy (available in a subgroup of 52 patients)	The overall prevalence of NAFLD was 47%. The prevalence of NASH was 22% in those who underwent liver biopsy. Psoriatic patients with NAFLD had higher mean PASI scores than those without NAFLD
Candia *et al.* 2015 [27]	Systematic review and meta-analysis: 7 case-control studies included	Ultrasonography and liver enzymes	Psoriatic patients had an increased risk of prevalent NAFLD compared with control subjects (6 studies, *n* = 267,761 patients, OR 2.15, 95% CI 1.6–2.9, *p* < 0.05). The risk of prevalent NAFLD was higher in patients with psoriatic arthritis (3 studies, *n* = 505 patients, OR 2.25, 95% CI 1.4–3.7, *p* < 0.05) and in those with moderate-to-severe psoriasis compared with patients with mild psoriasis (2 studies, *n* = 51,930 patients, OR 2.07, 95% CI 1.6–2.7, *p* < 0.05)

Abbreviations: ALT, alanine aminotransferase; AST, aspartate aminotransferase; BMI, body mass index; CI, confidence interval; NAFLD, nonalcoholic fatty liver disease; NASH, nonalcoholic steatohepatitis; PASI, psoriasis area and severity index; OR, odds ratio.

For instance, in a case-control study involving 130 consecutive patients with chronic plaque psoriasis (none of whom treated with methotrexate or other potentially hepato-toxic drugs) and 260 matched healthy controls, Gisondi *et al.* [19] have documented that NAFLD prevalence was almost two times higher among psoriatic patients than among control individuals (47% *vs.* 28%, *p* < 0.001). This difference remained significant (37% *vs.* 21%; *p* < 0.01), even after excluding subjects with mild-moderate alcohol consumption (*i.e*., those who drank less than 30 grams of alcohol per day). Patients with psoriasis and NAFLD were also more likely to have higher circulating levels of C-reactive protein, IL-6 and lower adiponectin levels than those without NAFLD. Furthermore, NAFLD was associated with a greater clinical severity of psoriasis as estimated by the Psoriasis Area and Severity Index (PASI) score after adjusting for many cardio-metabolic risk factors [19]. This score measures the severity of psoriatic lesions (evaluating the degree of erythema, thickness, and scaling of psoriatic plaques in four separate body areas) based on area coverage and plaque appearance.

In another retrospective study Miele *et al*. [20] found a NAFLD prevalence of 59.2% in an outpatient cohort of 142 adults with psoriasis. Although there were no differences in PASI score between psoriatic patients with or without NAFLD, those with NAFLD were more likely to have psoriatic arthritis and more severe NAFLD as estimated non-invasively with the NAFLD fibrosis score. Unfortunately, data on liver biopsy were available only for five psoriatic patients, but revealed that three of these patients had histologically proven NASH.

Interestingly, in a large population-based cohort study which included 2292 elderly individuals of whom 5.1% had psoriasis, van der Voort *et al.* [22] documented that the prevalence of NAFLD on ultrasonography was greater among psoriatic patients than among the reference group without psoriasis (46.2% *vs.* 33.3%, *p* = 0.005). Notably, multivariate regression analysis revealed that psoriatic participants were 70% more likely to have NAFLD than those without psoriasis (odds ratio (OR) 1.70, 95% confidence interval (CI) 1.1–2.6, *p* = 0.01), independent of metabolic syndrome and other common NAFLD risk factors. In a subsequent analysis of the same cohort, the authors have also reported that the prevalence of advanced hepatic fibrosis, as detected by transient elastography, was greater among those with psoriasis than among those without this disease (8.1% *vs.* 3.6%, *p* < 0.05), and that psoriatic patients were twice as likely to have advanced hepatic fibrosis, irrespective of common risk factors (adjusted-OR 2.57, 95% CI 1.0–6.6) [23]. Similarly, in a smaller case-control study, Gisondi *et al.* [24] reported that the NAFLD fibrosis score (*i.e*., a non-invasive scoring system that identifies advanced hepatic fibrosis) was higher in psoriatic patients than in control subjects, and psoriasis predicted advanced liver fibrosis, independently of coexisting metabolic syndrome features and other potential confounding factors.

Recently, in a cross-sectional study involving 103 United States middle-aged adult patients with psoriasis or psoriatic arthritis recruited over a 24-month period, Roberts *et al.* [26] found that the prevalence of ultrasound-diagnosed NAFLD was 47%, whereas that of NASH was 22% among those (*n* = 52) who underwent liver biopsy. Moreover, similarly to previous studies, the authors also found that psoriatic patients with NAFLD had significantly higher PASI scores than those without this disease.

Finally, a recent systematic review and meta-analysis of seven case-control studies confirmed that psoriatic patients had a two-fold increased rate of prevalent NAFLD compared with non-psoriatic control individuals, and that this risk was higher among those with either more severe psoriasis or psoriatic arthritis. Interestingly, the significant relationship between psoriasis and NAFLD was consistent in all studies included in this meta-analysis and was maintained even when the studies of lower methodological quality (due to poorly documented diagnosis of NAFLD or insufficient adjustment for potential confounding variables) were excluded from the analysis [27]. However, it is important to note that the cross-sectional nature of the above-mentioned studies does not permit to ascertain the temporality and causality of the association between NAFLD and psoriasis [19,20,21,22,23,24,25,26,27]. Future follow-up studies are required to improve our understanding of this topic.

That said, the data available to date show that NAFLD prevalence is very high in patients with psoriasis (affecting up to 50% of these patients), independent of coexisting metabolic syndrome components. In addition, the relatively advanced stage of NASH revealed by the biopsies from psoriatic patients suggests the possibility of an increased risk of long-term liver-related complications in this patient population. Thus, the current evidence argues for more careful monitoring and evaluation of the presence of NAFLD in people with chronic plaque psoriasis.

## 4. Potential Biological Mechanisms Linking Psoriasis and NAFLD

To date, the underlying mechanisms linking NAFLD to psoriasis are complex and not fully understood. However, identification of the pathophysiological mechanisms linking these two diseases is of clinical relevance because it may offer the promise for novel pharmacological approaches.

Psoriasis and NAFLD share multiple inflammatory and cytokine-mediated mechanisms and are part of an intriguing network of genetic, clinical and pathophysiological features. Indeed, it is possible to assume that the mechanisms underlying the association between NAFLD and psoriasis are multifactorial (involving both genetic and environmental factors) and often overlap with metabolic abnormalities, which frequently coexist in psoriatic patients.

**Figure 2:**
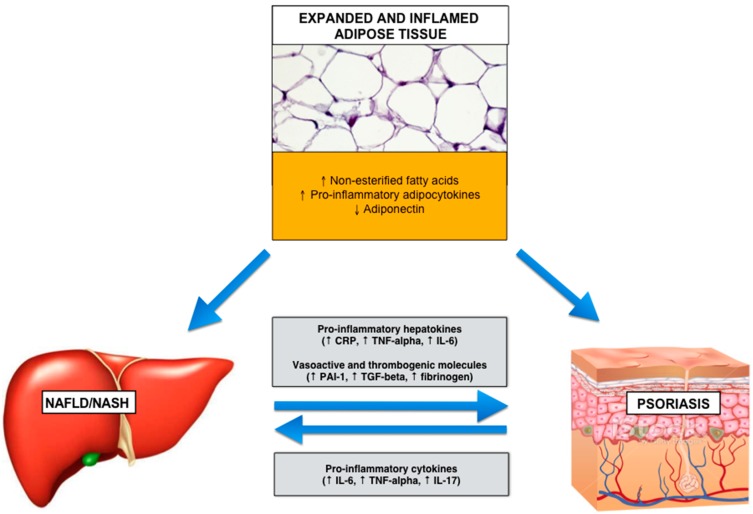
Possible mechanisms linking expanded and inflamed (dysfunctional) visceral adipose tissue, psoriasis and nonalcoholic fatty liver disease. Abbreviations: CRP, C-reactive protein; IL-6, interleukin-6; IL-17, interleukin-17; NAFLD, nonalcoholic fatty liver disease; NASH, nonalcoholic steatohepatitis; PAI-1, plasminogen activator inhibitor-1; TGF-β, transforming growth factor-β; TNF-α, tumor necrosis factor-α.

The schematic Figure 2 shows the possible links between expanded visceral adipose tissue, steatotic liver and psoriatic skin, and the signals passing between these three organs.

Although the liver is a key regulator of glucose metabolism, and is the leading source of multiple inflammatory and coagulation factors [5,9,28], the close inter-relationships of psoriasis and NAFLD with visceral obesity and insulin resistance make it very difficult to distinguish the individual contribution of NAFLD to the inflammatory and metabolic manifestations of psoriasis. Although the studies available in the literature do not allow to clearly determine the directionality of the association between NAFLD and psoriasis, it is conceivable that several pro-inflammatory cytokines (e.g., IL-6, IL-17, TNF-α) that are locally over-produced by lymphocytes and keratinocytes into the skin of psoriatic patients may contribute, at least in part, to the pathogenesis of systemic insulin resistance [29,30], and that psoriatic patients with greater insulin resistance are the ones who get NAFLD. Undoubtedly, an expanded and inflamed (dysfunctional) visceral adipose tissue plays a key role in the development of insulin resistance, chronic inflammation and NAFLD, possibly through the secretion of multiple factors, such as increased release of non-esterified fatty acids, increased production of various hormones and pro-inflammatory adipocytokines (including also TNF-α, IL-6, leptin, visfatin, and resistin), and decreased production of adiponectin [9,31,32,33,34]. In the presence of obesity and insulin resistance, there is an increased influx of non-esterified fatty acids to the liver. There is now substantial evidence that non-esterified fatty acids play a key role in directly promoting liver injury by increasing intra-hepatic oxidative stress and by activating inflammatory pathways [9,31,32,33,34]. The central role of hepatocyte cytokine production in NAFLD progression is supported by studies showing that cytokines may replicate all of the histological features associated with NASH, including neutrophil chemotaxis, hepatocyte necrosis and stellate cell activation [9,31,32,33,34]. It is possible to assume that the increased release of non-esterified free fatty acids from the expanded and dysfunctional adipose tissue, in presence of insulin resistance, may also exert a deleterious impact on inflammatory skin lesions in psoriasis. However, to our knowledge, there are currently no reliable data regarding a direct pathogenic role of non-esterified fatty acids in the pathogenesis of psoriasis. Further studies are required to better elucidate this topic.

To date, accumulating evidence indicates that NAFLD, especially its necro-inflammatory and progressive form (NASH), may exacerbate insulin resistance, predisposes to atherogenic dyslipidemia and releases a myriad of pro-inflammatory, pro-coagulant, pro-oxidant and pro-fibrogenic mediators (e.g., C-reactive protein, IL-6, fibrinogen, plasminogen activator inhibitor-1, transforming growth factor-β) that may play important roles in the pathophysiology of psoriasis [5,9,31,35]. It is possible to hypothesize that the release of these pro-inflammatory, pro-oxidant and pro-atherogenic mediators from the steatotic and inflamed liver (which is also one of the most important mechanisms by which fatty liver directly contributes to the development of cardiovascular disease and type 2 diabetes [5,9,36]) may adversely influence the severity of psoriasis by increased keratinocyte proliferation, increased inflammation, and up-regulation of various vascular adhesion molecules. Experimentally, it has been also shown that induction of oxazolone-induced skin inflammation is more evident in NAFLD mice than in normal mice; oxazolone challenge significantly increases ear thickness, ear weight, nuclear factor-κB activity, and histological features of skin inflammation in NAFLD mice as compared to normal mice [37]. The oxazolone-induced skin inflammation model is not specifically designed to study the pathogenesis of psoriasis. Nevertheless, this simple mouse model of NAFLD-enhanced skin inflammation might be used to evaluate new therapeutic strategies for treatment of NAFLD with associated skin inflammation and also to understand the nexus between these two co-morbidities.

## 5. Treatment for Psoriasis and Its Potential Implications for NAFLD

Detailed discussion of treatment options for psoriasis is beyond the scope of this review and have been recently discussed elsewhere [38]. There are numerous treatment options against psoriasis and they are classified as topical, systemic or phototherapy. Systemic drugs such as methotrexate, cyclosporine and acitretin are indicated for moderate-to-severe psoriasis, especially when the disease is either widespread or resistant to topical therapy. In the case of intolerance, inefficacy or contraindication to either phototherapy or conventional systemic treatments, patients with psoriasis are eligible for newer biological agents, which include TNF-α antagonists (etanercept, adalimumab and infliximab), the anti-IL-2/23 monoclonal antibody ustekinumab, and the anti-IL-17 monoclonal antibodies secukinumab and ixekizumab [38].

From a clinical perspective, understanding whether psoriatic patients have underlying metabolic comorbidities, including NAFLD, is important to ensure that treatment is safe [38,39]. Indeed, while phototherapy or topical treatments are not expected to cause significant changes in metabolic parameters and liver function tests, some pharmacological treatments may negatively influence metabolic comorbidities (including NAFLD) or exert interactions with drugs that are commonly used to treat them [39].

In particular, methotrexate should be administered with caution in the presence of obesity, type 2 diabetes or NAFLD because of the increased risk of drug-induced hepatic fibrosis [40,41,42]. Indeed, psoriatic patients with type 2 diabetes or obesity are at higher risk of developing hepatic fibrosis during methotrexate treatment compared with those without such metabolic comorbidities [39]. The liver injury induced by methotrexate appears to mimic NAFLD histologically. So, drug induced liver injury should be always considered in a patient with hepatic steatosis who has been previously treated with methotrexate [40,41]. Similarly, cyclosporine should be used cautiously among psoriatic patients with coexisting metabolic syndrome. This drug may worsen type 2 diabetes, exacerbate arterial hypertension and predispose to atherogenic dyslipidemia and hyperuricemia [38,43]. Moreover, the drug interaction between cyclosporine and statins may also increase the risk of rhabdomyolysis [44]. In some cases, cyclosporine may induce liver injury and cholestasis with increased levels of serum aminotransferases, bilirubin and alkaline phosphatase [38,43]. However, cyclosporine-induced hepatitis is a relatively rare event that is less common than nephrotoxicity and occurs more frequently among liver-transplant patients. Acitretin is a vitamin A derivative that has been used to treat psoriasis since the early 1980s. The use of acitretin is limited by its potential adverse effects (e.g., muco-cutaneous effects, dyslipidemia and hepatotoxicity). These effects may be reduced by using lower doses of acitretin or in combination with other therapies [43,45].

Biologic drugs represent a major advancement in the treatment of psoriasis [38]. Generally, biologic agents do not seem to negatively affect metabolic parameters and serum liver enzyme levels as conventional systemic treatments can. Indeed, the drug survival of biologics is higher than that of conventional treatments because they are better tolerated in the longer term. Although the effects of TNF-α inhibitors on insulin sensitivity are a matter of intense debate [38,43,46], preliminary evidence suggests that treatment with etanercept (*i.e*., a TNF-α inhibitor) may improve both plasma glucose levels and insulin resistance indices [47], and that patients with psoriasis or rheumatoid arthritis receiving TNF-α inhibitors exhibit a lower risk of new-onset type 2 diabetes compared with those receiving other non-biological disease-modifying anti-rheumatic drugs [48]. Clinically meaningful dyslipidemia has been rarely reported in patients receiving etanercept or other TNF-α antagonists, so that it is not a serious concern in routine clinical practice [49]. A significant body weight gain, mainly due to increased fat mass, has been also documented among psoriatic patients receiving TNF-α antagonists [38,39,43], whereas it is not observed among those receiving the anti-IL-12/23 monoclonal antibody ustekinumab [50]. Mild to moderate elevations in serum transaminases may be observed in some patients receiving TNF-α antagonists (especially infliximab [51]), but they usually return to normal after discontinuation of the drug [38,43,52]. In a small clinical trial, Campanati *et al.* [53] have recently compared the effect of a 24-week treatment with etanercept *versus* phototherapy on serum markers of hepatic fibrosis in 89 overweight patients with psoriasis and NAFLD. Notably, they found that there were significant improvements in the aspartate aminotransferase-to-alanine aminotransferase ratio, serum C-reactive protein levels and insulin resistance indices only among psoriatic patients receiving etanercept. This finding suggests that etanercept is more efficacious to reduce the risk of hepatic fibrosis than phototherapy, and that this effect might be mainly dependent on its metabolic and anti-inflammatory properties. However, additional studies with more accurate and direct measures of hepatic fibrosis are needed to further examine this topic. Recently, preliminary evidence has suggested that NAFLD might also be a side effect of TNF-α inhibitor treatment in some cases, and that previous methotrexate exposure and patatin-like phospholipase domain-containing protein-3 (PNPLA3) genotype might be the most important risk factors [54]. Even though only few cases have been reported in the literature, TNF-α inhibitors may induce autoimmune hepatitis, granulomatous hepatitis, and reactivation of viral hepatitis [38,52].

Finally, similarly to patients with NAFLD, lifestyle interventions (hypocaloric diet, exercise and avoiding alcohol consumption) are the mainstay treatment for the majority of psoriatic patients because they may also improve the response to pharmacological treatments for psoriasis [38,39,43]. It is known that the risk of psoriasis and its clinical severity are closely associated with the degree of overweight/obesity of this patient population. Although weight loss alone may be insufficient for maintaining skin disease remission in obese patients with psoriasis [55], some recent intervention trials have demonstrated that treatment with a low-energy diet showed a trend towards significant improvement in PASI scores among overweight or obese patients with psoriasis, and that body weight reduction in psoriatic patients receiving either low-dose cyclosporine or biologics increased the efficacy of these drugs [56,57,58]. A recent systematic review and meta-analysis including four small randomized clinical trials with either pioglitazone or rosiglitazone that examined the efficacy of glitazones on psoriasis severity has concluded that pioglitazone may exert some positive effect on psoriasis [59]. However, the clinical significance of this effect and role of this drug in management of psoriatic patients deserve further study.

There are as yet few proven therapies available for patients with NAFLD and NASH, and current therapeutic strategies are specifically directed towards improving features of the metabolic syndrome [5,9,36]. Pioglitazone has the best evidence-based data for NASH treatment. To date, however, lifestyle changes are the more effective therapeutic option that is sharable between patients with NAFLD and those with psoriasis. To our knowledge, no randomized clinical trials have specifically examined the effects of chronic treatment with the newer biologic agents on histologic features of NAFLD. Therefore, additional studies are required to evaluate the best approach to management of NAFLD among patients with psoriasis.

## 6. Conclusions

Although the published evidence is restricted to observational (cross-sectional and case-control) studies [17,18,19,20,21,22,23,24,25,26,27], a growing body of clinical evidence suggests a strong relationship between NAFLD and psoriasis. Published studies indicate that NAFLD is a very frequent condition among adult patients with psoriasis (affecting up to 50% of these patients) and that patients with psoriasis and NAFLD are more likely to have metabolic syndrome and a more severe degree of skin disease than their counterparts without NAFLD. In addition, psoriatic patients are at higher risk of developing the more severe forms of NAFLD (*i.e*., about a quarter of these patients may develop NASH during the course of the disease). However, further research is required to ascertain whether NAFLD is merely an epiphenomen of coexisting metabolic syndrome features, or is an independent risk factor for the development and progression of psoriasis. Additional studies are also needed to better elucidate the putative biological mechanisms linking NAFLD with psoriasis. Specific mediators of this novel “hepato-dermal axis” need to be further investigated in order to discover innovative drugs and treatments.

In the meantime, given the strong association between NAFLD and psoriasis, we believe that health care providers following psoriatic patients should be mindful of this potentially progressive liver disease that is commonly observed among psoriatic patients. The presence of NAFLD should be also taken into consideration when choosing pharmacological treatment, as some conventional drugs for psoriasis are potentially hepatotoxic.

These findings imply that psoriatic patients should be routinely screened for NAFLD and that consideration should be given to referring these patients to a hepatologist for further evaluation. The optimal method of screening is presently unknown. However, given the intrinsic limitations of serum liver enzyme levels as initial screening test for NAFLD, we think liver ultrasound and transient elastography combined with the use of the NAFLD fibrosis score or other non-invasive fibrosis scoring systems are useful as first-line options in identifying patients with suspected NASH to submit to biopsy among those with psoriasis [60,61,62]. Moreover, all these patients should be followed regularly to monitor the development of liver-related, metabolic and cardiovascular complications [63].
